# An Interpretable Ensemble Machine Learning Framework for Predicting the Ultimate Flexural Capacity of BFRP-Reinforced Concrete Beams

**DOI:** 10.3390/polym18050601

**Published:** 2026-02-28

**Authors:** Sebghatullah Jueyendah, Elif Ağcakoca

**Affiliations:** Department of Civil Engineering, Sakarya University, Esentepe Campus, Serdivan 54050, Sakarya, Türkiye; sebghatullahjueyendah@gmail.com

**Keywords:** basalt fiber-reinforced polymer, reinforced concrete beams, ultimate flexural capacity, machine learning, ensemble learning, structural engineering, explainable artificial intelligence, SHAP

## Abstract

Prediction of the ultimate moment capacity (Mu) of BFRP-reinforced concrete beams is complicated by nonlinear parameter interactions and the linear-elastic response of BFRP, reducing the accuracy of conventional design models. This study develops an optimized machine learning (ML) framework incorporating random forest, extra trees, gradient boosting, adaboost, bagging, support vector regression, histogram-based gradient boosting, and ensemble voting and stacking strategies for reliable prediction of the Mu of BFRP-reinforced concrete beams. A comprehensive database of material, geometric, reinforcement, and BFRP mechanical parameters was analyzed, and model performance was evaluated using an 80/20 train–test split and 10-fold cross-validation based on R^2^, RMSE, MAE, and MAPE. The stacking regressor demonstrated superior predictive performance, achieving an R^2^ of 0.999 (RMSE = 0.590) in training and an R^2^ of 0.988 (RMSE = 2.487) in testing, indicating excellent robustness and strong generalization capability in predicting Mu. Furthermore, interpretability analyses based on SHAP, PDP, ALE, and ICE demonstrate that span length (L) and beam depth (h) constitute the governing parameters in the prediction of Mu. Unlike prior studies focused mainly on predictive accuracy, this work proposes an optimized and interpretable stacking ensemble framework that integrates explainable AI with classical flexural mechanics for physically consistent and reliable prediction of the ultimate moment capacity of BFRP-reinforced concrete beams.

## 1. Introduction

Steel reinforcement corrosion remains a major durability concern in reinforced concrete structures exposed to aggressive environments [1]. Driven by corrosion-related deterioration of steel reinforcement and their favorable mechanical and durability properties, the use of fiber-reinforced polymer (FRP) bars as internal reinforcement in concrete structures has increased substantially in recent decades [2]. Among FRP reinforcement types, basalt fiber-reinforced polymer (BFRP) bars have attracted increasing attention as cost-effective, corrosion-resistant alternatives to steel reinforcement, owing to their favorable mechanical properties, chemical stability, and resistance to alkaline environments [3]. The linear-elastic, brittle behavior and lower elastic modulus of BFRP bars limit the applicability of conventional steel-based design models and complicate the accurate prediction of Mu capacity [4]. Consequently, the reliable prediction of the Mu capacity of BFRP-reinforced concrete beams remains an open and challenging research problem [5]. Although existing design codes adopt conservative provisions to ensure safety, such conservatism may result in uneconomical designs, despite valuable insights gained from experimental investigations on the flexural behavior of BFRP-reinforced concrete beams. Experimental and analytical approaches are limited by cost and simplifying assumptions, prompting the adoption of ML techniques to model complex nonlinear behavior directly from data [6]. Conventional empirical and regression-based models often fail to adequately capture complex nonlinear relationships, thereby limiting their predictive accuracy and practical applicability [7]. Recent advances in artificial intelligence (AI), ML, and deep learning (DL) have enabled data-driven modeling of complex interactions and are increasingly being adopted in civil and structural engineering applications [8]. In 1959, Arthur Samuel defined ML as a system’s ability to learn from experience and enhance its performance without explicit programming [9]. AI’s prominence arises from its ability to model complex phenomena and predict outcomes, with ML facilitating pattern recognition and data-driven decision-making [10]. Researchers increasingly employ ML to predict concrete strength and enhance civil and structural engineering applications [11,12]. Hassan et al. [13] demonstrated that ML techniques can effectively predict the shear strength of FRP-reinforced concrete beams. Karabulut [14] employed ML regression models to predict the flexural performance of GFRP bar–reinforced concrete beams with high accuracy. Tran et al. [15] presented a comprehensive state-of-the-art review on the flexural behavior, design approaches, and limitations of FRP-reinforced concrete beams. Rahman and Al-Ameri [16] developed ANN-based models to predict the flexural performance of BFRP-reinforced self-compacting geopolymer concrete beams. Zhang et al. [17] developed an ML-based capacity prediction model for CFST columns with damaged BFRP jackets and employed SHAP analysis to identify key influencing parameters. Hasan et al. [18] predicted the compressive strength of hybrid-fiber-reinforced recycled aggregate concrete using ML, providing a robust tool for mix design optimization. Dahish and Alkharisi [19] applied ML and response surface methodology to predict properties of polypropylene fiber recycled aggregate concrete, showing ML as a sensitive predictor, particularly for tensile properties. Uysal and Tanyildizi [20] evaluated the compressive strength of polypropylene FRSCC under high temperatures. Jueyendah et al. [21] showed that SVR with an RBF kernel effectively predicts cement mortar strength. Jueyendah and Martins [22] developed a hybrid SVR-RBF model for optimized design and performance assessment of welded structures. Mashhadban et al. [23] modeled and predicted the mechanical properties of FRSCC using particle swarm optimization and ANN. Jueyendah et al. [24] demonstrated that nonlinear ML models provide superior accuracy compared to linear models in predicting cement mortar strength. de Carvalho et al. [25] developed an ML-based design approach for concrete-filled stainless steel tubular columns. Hematibahar et al. [26] proposed an SADRA-based ML model to predict fiber-reinforced concrete. Song et al. [27] developed a novel self-compacting ultra-high-performance fiber-reinforced concrete using compounded high-activity powders. Begum et al. [28] investigated the effect of steel fibers on the properties of fly ash-based SCC. Saha et al. [29] predicted the strength of SCC using ANN and multivariable regression analysis. Pons et al. [30] investigated the mechanical behavior of self-compacting concrete reinforced with hybrid fibers. Ağcakoca et al. [31] proposed a hybrid ML–FE model using CDP to enhance prediction of cementitious composite behavior. Siddique et al. [32] modeled and predicted the CS of SCC using ANN. Maabreh and Almasabha [33] utilized ML techniques to evaluate and predict the shear strength of deep steel fiber-reinforced concrete beams. Megahed et al. [34] utilized ML models to predict the load-bearing capacity of concrete-filled steel tubular columns. Fakharian et al. [35] predicted the CS of green concrete incorporating recycled glass-fiber-reinforced polymers using a ML approach. ML has been widely used to predict reinforced concrete behavior, yet studies on BFRP-reinforced beams remain limited, and existing models often function as low-interpretability black boxes, restricting their practical adoption. In addition to ML-based approaches, experimental and theoretical investigations provide fundamental insights into structural behavior, informing both feature selection and the interpretation of predictive models. Zeng et al. [36] reported that FRP grid-reinforced ultra-high-performance concrete (UHPC) plates incorporating 12 mm polyethylene (PE) fibers at a 1% volume fraction exhibit the most efficient and cost-effective enhancement of flexural performance, achieving increases in capacity of up to 200%. Fan et al. [37] showed that FRP-reinforced UHPC tubular beams with steel or PE fibers exhibit comparable fatigue life, with steel fibers improving post-cracking stiffness, and recommend a 50% ultimate load endurance limit for design. Zeng et al. [38] assessed the durability of FRP grid-reinforced UHPC plates in marine environments, highlighting their superior stiffness and resistance compared to conventional FRP-reinforced concrete. Liao et al. [39] examined FRP grid-reinforced UHPC pipes under lateral compression, demonstrating that internal FRP grids significantly enhance the structural performance of UHPC pipes. Jafari et al. [40] showed that beam geometry, FRP stirrup properties, and concrete strength critically influence the performance of FRP-RC beams, highlighting the importance of both material and geometric parameters in predictive modeling. Shahmansouri et al. [41] showed that ML models accurately capture the bond–slip behavior of steel rebars in CFRP-confined low-strength concrete, with compressive strength and rebar diameter as key factors. Experimental insights into cracking and reinforcement–matrix behavior justify key features and support the nonlinear interactions identified by SHAP and ALE, aligning ML predictions with classical flexural mechanics. To overcome these limitations, this study presents a novel interpretable ML framework for predicting the Mu of BFRP-reinforced concrete beams. A variety of state-of-the-art individual, ensemble, and hybrid ML models are developed, evaluated, and compared under consistent criteria, while explainable AI techniques quantify feature importance and clarify key parameter influences. The proposed framework improves predictive accuracy and ensures model transparency, thereby enhancing design reliability, mitigating conservatism, and offering a practical decision-support tool for performance-based design and optimization of FRP-reinforced concrete structures. Unlike previous ML studies that emphasize algorithm performance, this work presents an interpretable framework linking data-driven predictions with classical structural mechanics. A systematically optimized stacking ensemble is integrated with multi-level explainable AI (SHAP, PDP, ALE, ICE) and validated against flexural equilibrium and strain compatibility principles. By combining optimization, interpretability, and mechanics-based validation, this study delivers a physically consistent and practically applicable framework for predicting the ultimate moment of BFRP-reinforced concrete beams.

## 2. Materials and Methods

A comprehensive database of 220 BFRP-reinforced concrete beam specimens was compiled from published studies [42,43,44,45,46,47,48,49,50,51,52,53,54,55,56,57,58,59,60,61,62,63,64]. The present study considers key input parameters, including concrete compressive strength (CS), beam width (b), beam depth (h), effective depth (d), span length (L), shear span (a), reinforcement ratio (ρ), BFRP bar diameter (dBFRP), BFRP elastic modulus (EBFRP), and BFRP tensile strength (Fu, BFRP), with the Mu capacity designated as the target output variable. Random forest (RF), extra trees (ET), gradient boosting (GB), adaboost (AB), bagging (BG), support vector regression (SVR), and histogram-based gradient boosting (HGB) models, along with advanced voting regressor (VR) and stacking regressor (SR) ensemble strategies, were applied to maximize predictive performance and robustness in estimating the Mu of BFRP-reinforced concrete beams. The dataset was partitioned into training and testing subsets using an 80/20 split, with model hyperparameters optimized via grid search, and predictive performance and robustness assessed through R^2^, RMSE, MAE, MAPE, alongside 10-fold cross-validation, while statistical analyses were conducted to evaluate data distribution and variability. Model interpretability was examined through comprehensive explainability analyses, employing SHAP values, partial dependence plots (PDPs), accumulated local effects (ALE), and individual conditional expectation (ICE) plots to elucidate the influence of key input features. A comprehensive and methodologically rigorous data screening procedure was conducted to ensure the validity, consistency, and overall integrity of the assembled dataset. An initial dataset comprising 220 experimental specimens obtained from the published literature was subjected to a rigorous validation procedure. Records exhibiting incompleteness or inconsistency were systematically eliminated, resulting in a curated dataset of 208 qualified samples encompassing all requisite engineering parameters for subsequent analytical evaluation. Outlier detection was conducted using the Z-score method and interquartile range (IQR) analysis, confirming the absence of extreme anomalies and ensuring statistical consistency. Furthermore, the Kolmogorov–Smirnov test was employed to evaluate distributional characteristics, and, in conjunction with the verified parameter coverage, confirmed the robustness and representativeness of the established database. Table 1 summarizes descriptive statistics, Kolmogorov–Smirnov normality tests, and VIF values for the input features. After normalization and outlier elimination via the Z-score and IQR methods, VIF and KS test statistics (*p*-values) were evaluated for all variables. CS and Mu exhibit moderate variability, whereas geometric parameters (b, h, d, L, and a) show comparatively greater dispersion. The KS test indicated that most variables deviate from strict normality (*p* < 0.05); however, this does not limit the applicability of non-parametric ensemble ML models. Furthermore, VIF analysis revealed severe multicollinearity among geometric variables (VIF > 90), whereas material-related parameters (CS, ρ, dBFRP, EBFRP, and Fu, BFRP) exhibited acceptable VIF values (≈1–1.7), indicating negligible collinearity.

**Figure 1:**
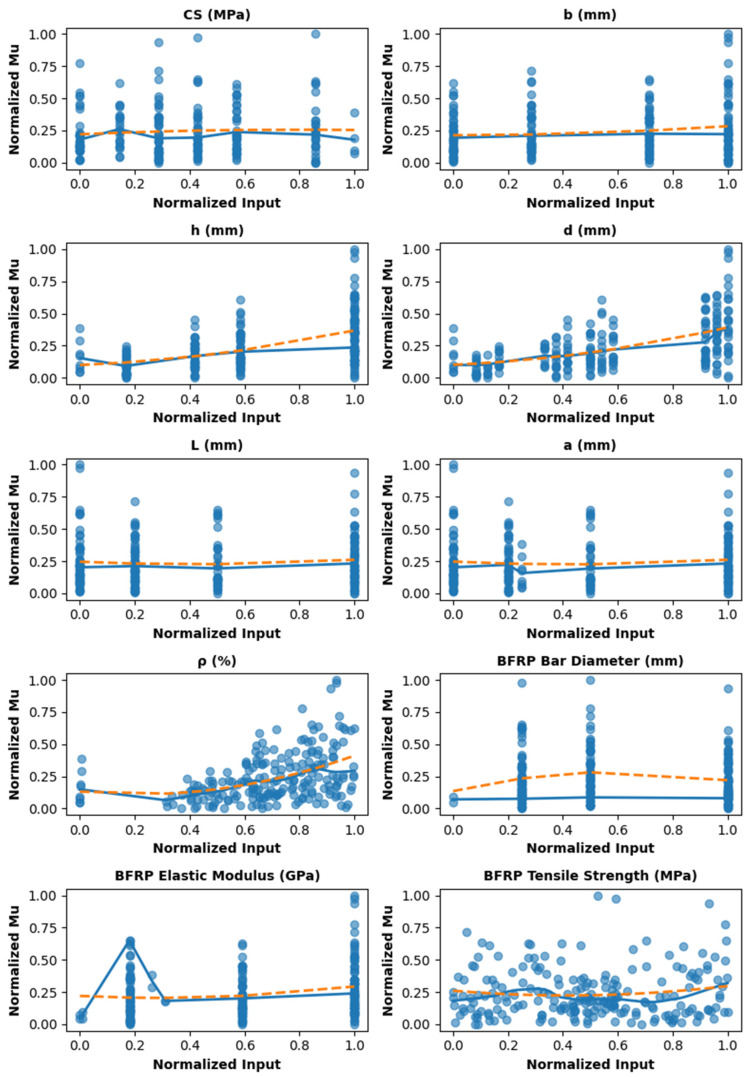
Nonlinear relationship between input parameters and Mu.

**Figure 2:**
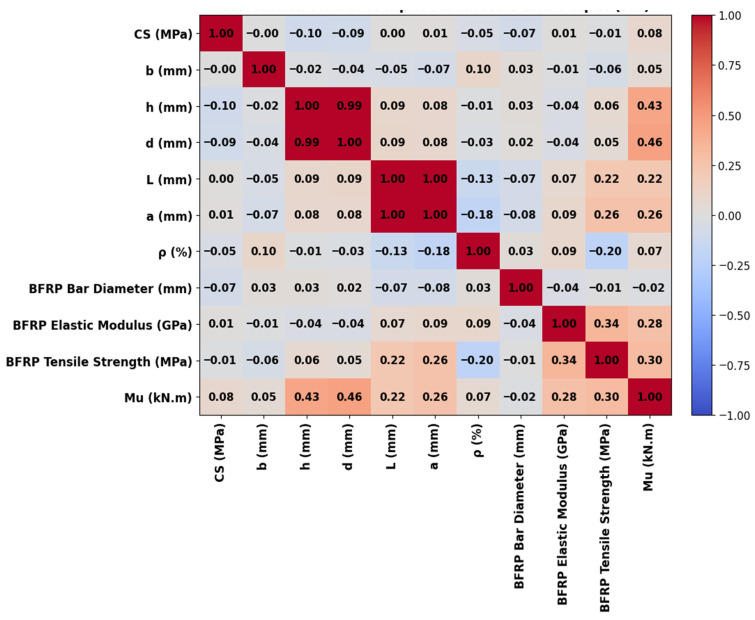
Correlation matrix of input variables and ultimate moment.

After normalization and outlier removal using the Z-score and IQR methods, statistical analyses were performed, including the computation of the variance inflation factor (VIF) and Kolmogorov–Smirnov (KS) test statistics. Additionally, a correlation heatmap was generated to examine linear relationships among the input and output variables (CS, b, h, d, L, a, ρ, dBFRP, EBFRP, Fu, BFRP, and Mu). VIF and the KS test were computed, yet they do not capture the inherently nonlinear interactions among structural parameters. Notably, VIF values above 10 indicate strong linear dependence, typically arising from physically correlated geometric features. Nevertheless, tree-based ensemble models remain robust to correlated predictors and non-normal distributions, enabling accurate modeling of complex nonlinear relationships. As illustrated in Figure 1, the relationships between the input parameters and the Mu exhibit pronounced nonlinearity. This observation indicates that traditional linear analyses, such as correlation coefficients and VIF, are insufficient to fully characterize these complex interactions. Consequently, the adoption of nonlinear modeling techniques, including tree-based ensemble ML methods, is warranted to enable more accurate prediction and comprehensive interpretation of the system behavior. Figure 2 presents the correlation matrix of key geometric, material, and reinforcement parameters for BFRP-reinforced concrete beams with respect to Mu. Mu correlates most strongly with beam depth (h, 0.43), effective depth (d, 0.46), and BFRP properties (elastic modulus 0.28, tensile strength 0.30), emphasizing the dominant role of geometry and reinforcement in flexural capacity. Other variables, including concrete compressive strength (CS, 0.08), beam width (b, 0.05), reinforcement ratio (ρ, 0.07), and BFRP diameter (−0.02), exhibit weak linear correlations with Mu. Beam depth and effective depth (h and d, 0.99), and span length and shear span (L and a, 1.00), are strongly inter-correlated, reflecting structural dependencies.

**Figure 3:**
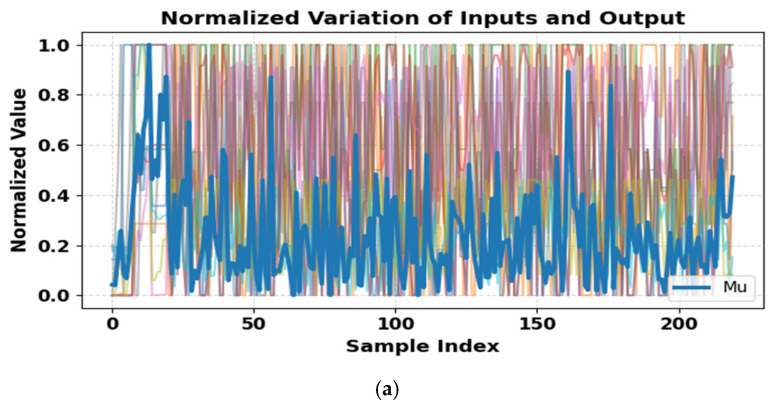

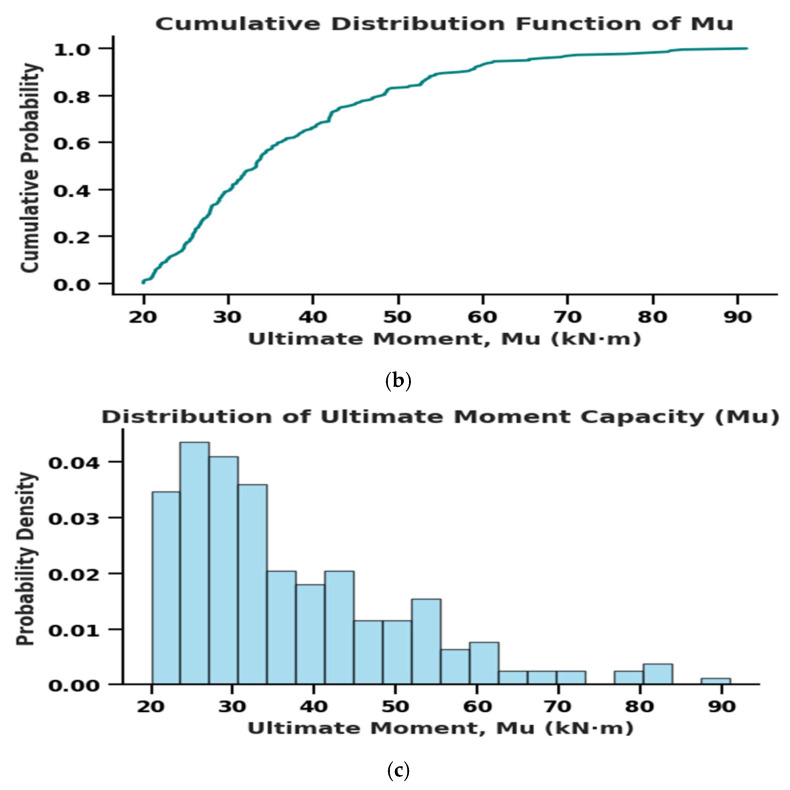
Variation and distribution of ultimate moment capacity with normalized input parameters.

Figure 3 illustrates the variation and distribution of ultimate Mu with normalized input parameters. Figure 3a shows normalized inputs and Mu, highlighting their relative magnitudes and variability; Figure 3b presents the CDF, indicating most beams have Mu below 50 kN·m; and Figure 3c depicts the positively skewed PDF with concentrations between 25–40 kN·m. Overall, the figure captures the variability, central tendency, and spread of Mu across the dataset. Data analysis and model development were conducted in Python 3.11 using an integrated development environment, enabling efficient preprocessing, training, and evaluation. Python, supported by scientific libraries such as NumPy and Pandas, provided a robust and efficient computational environment for data processing, model development, evaluation, and visualization in this study [65,66]. The dataset was preprocessed, and features were engineered to capture key physical relationships affecting Mu.

### 2.1. Machine Learning Models for Mu Prediction

Random forest (RF) and extremely randomized trees (ET) mitigate model variance by aggregating the predictions of multiple decision trees, thereby providing robust and reliable predictions of the Mu [67]. Gradient boosting (GB), adaboost (AB), and histogram-based gradient boosting (HGB) sequentially enhance predictive performance by iteratively correcting errors from preceding models, thereby capturing complex nonlinear relationships in beam behavior [68]. Bagging (BG) enhances predictive reliability by training base learners on different data subsets, while support vector regressor (SVR) effectively captures nonlinear patterns, particularly in smaller datasets [69]. Voting regressor (VR) combines multiple models to exploit their complementary strengths, and stacking regressor (SR) employs a meta-model to optimally integrate individual predictions, resulting in improved predictive accuracy [70]. Bagging methods primarily reduce variance, boosting methods focus on bias reduction, and stacking improves model generalization by optimally combining multiple learners. These ml models are essential in structural engineering for precise and reliable Mu estimation. These approaches enhance the accuracy and robustness of ultimate moment capacity predictions, thereby supporting safe and efficient beam design [71]. The selected learning algorithms are designed to model the nonlinear, interaction-dominated behavior of Mu in BFRP-reinforced beams while maintaining robustness for a moderate-sized dataset. Tree-based ensemble methods were utilized for their capability to accurately represent complex geometric–material interactions and their intrinsic robustness to experimental uncertainties [72]. Gradient boosting techniques facilitate the modeling of higher-order nonlinearities in demand–capacity relationships, while variance-reduction strategies enhance model stability across heterogeneous experimental datasets [73]. SVR captures smooth, physics-consistent flexural behavior, while voting and stacking ensembles improve robustness and generalization by reducing bias and variance in noisy, moderate-sized datasets [74].

### 2.2. SHAP-Based Interpretability

SHapley Additive exPlanations (SHAP) provide a rigorous and transparent interpretation of tree-based models by decomposing predictions into additive feature contributions based on cooperative game theory [75]. SHAP summary plots rank input variables by their global influence on CS while revealing the direction and magnitude of their effects. SHAP dependence plots depict the influence of individual features across their value ranges and reveal interaction effects, whereas SHAP dependency analysis quantifies higher-order interactions and evolving feature contributions. SHAP-based and traditional feature importance analyses improve model transparency by identifying the key factors controlling concrete strength and material behavior [76].

### 2.3. Partial Dependence Analysis

Partial dependence analysis (PDA), visualized through partial dependence plots (PDP), provides a rigorous assessment of the marginal influence of one or two input variables on the model response by averaging predictions across the joint distribution of all remaining features [77]. PDPs reveal nonlinear and threshold effects of inputs on predictions, providing robust global trend insights despite assuming feature independence. In civil engineering, PDPs facilitate identification of optimal material ranges, supporting performance-based mix optimization and informed design decisions [78].

### 2.4. Accumulated Local Effects Analysis

Accumulated local effects (ALE) plots provide a rigorous quantification of feature impacts by aggregating local variations in model predictions across the feature distribution, effectively accounting for feature correlations and minimizing extrapolation bias compared to PDP [79]. ALE plots provide unbiased, interpretable estimates of feature effects, particularly for complex datasets with correlated inputs. In civil engineering, ALE analysis provides precise insights into how material variations influence structural performance, thereby supporting robust decision-making in mix design and material selection [80].

### 2.5. Individual Conditional Expectation Analysis

Individual conditional expectation (ICE) plots illustrate the response of each sample to a specific feature, capturing heterogeneity in feature effects and delineating subgroups with distinct behavioral patterns [81]. They augment PDPs by uncovering local variations obscured by averaging, thereby improving the granular interpretability of model predictions. In structural engineering, ICE plots facilitate the assessment of how material variability influences individual concrete mixtures, thereby enhancing confidence in model predictions for specific design scenarios [82].

### 2.6. Statistical Parameters for Model Evaluation

Regression model performance is evaluated using multiple statistical metrics to ensure accuracy, reliability, and robustness [83]. R^2^ quantifies the proportion of variance in the dependent variable explained by the independent variables, with higher values indicating better predictive performance. RMSE and MAE measure the average deviation and absolute error between predicted and actual values, respectively, with lower values reflecting greater accuracy; MAE treats all errors equally, while RMSE penalizes larger deviations. MAPE assesses prediction accuracy as a percentage of actual values, providing a scale-independent measure of model performance. These metrics offer a robust framework for evaluating regression models in civil engineering [7]. The regression model’s performance was systematically evaluated using *R*^2^, *RMSE*, *MAE* and *MAPE* as defined in Equations (1)–(4).

(1) $$R^{2}=1-\;\frac{\sum_{i=1}^{n}{\left(y_{m,i}-y_{p,i}\right)}^{2}}{{\left(y_{m,i}-y_{a}\right)}^{2}}$$

(2) $$RMSE=\sqrt{\frac{1}{n}\sum_{i=1}^{n}{\left(y_{m,i}-y_{p,i}\right)}^{2}}$$

(3) $$MAE=\frac{1}{n}\sum_{i=1}^{n}\left|y_{m,i}-y_{p,i}\right|$$

(4) $$MAPE=\frac{100\%}{n}\sum_{i=1}^{n}\left|\frac{y_{m,i}-y_{p,i}}{y_{m,i}}\right|$$

The parameters *y_m_* and *y_p_* represent the actual and predicted values, respectively, while *y_a_* denote the mean of observed values used as a reference for model accuracy. The symbol *n* indicates the total number of samples in the dataset, and *p* refers to the number of independent input variables or predictors in the regression model. These parameters are used to evaluate the model’s predictive performance and statistical reliability.

## 3. Results and Discussion

Hyperparameters play a pivotal role in governing model complexity, bias–variance trade-offs, and overall predictive performance, as summarized in Table 2. In tree-based models (RF, ET, GB, AB, BG, HGB), hyperparameters like the number of estimators, tree depth, and learning rate balance accuracy and overfitting, while in SVR, C, epsilon, and kernel settings control its ability to capture non-linear relationships. In VR and SR models, hyperparameters of base and meta-models control prediction weighting and integration, enhancing robustness and generalization in Mu estimation.

Table 2 presents the hyperparameter configurations of the ML models for Mu prediction. Tree-based models, RF (n_estimators = 250, max_depth = 6), ET (n_estimators = 250, max_depth = 7), GB (n_estimators = 260, learning_rate = 0.1, max_depth = 3), AB (DT max_depth = 4, n_estimators = 300, learning_rate = 1.0), BG (DT, n_estimators = 245), and HGB (max_iter = 310, learning_rate = 0.1, max_depth = 6), were tuned to optimize accuracy and control overfitting. SVR (RBF kernel, C = 89, epsilon = 0.1) captured non-linear relationships. Ensemble models, VR (RF + ET + GB, n_estimators = 200 each) and SR (base: RF, GB, SVR; meta: linear regression), enhanced robustness and generalization. Initially, the features L, h, and BFRP tensile strength were excluded from the input set (Table 3), and subsequently incorporated (Table 4) to assess their influence on the predictive performance of the ML models for Mu.

Table 3 presents the training and testing performance of the ML models for predicting Mu without the inclusion of L, h, and BFRP tensile strength as input features. For each model, R^2^, RMSE, MAE, and MAPE are reported for training and testing datasets. SR achieved the highest predictive performance with R^2^ = 0.9934 (RMSE = 0.765, MAE = 0.678, MAPE = 1.564%) in training and R^2^ = 0.932 (RMSE = 3.313, MAE = 2.335, MAPE = 5.25%) in testing, demonstrating excellent generalization. Normalized root mean square error (NRMSE) quantifies prediction error relative to the magnitude of $M_{u}$, enabling scale-independent comparison among models. In training, gradient boosting achieved the lowest NRMSE (0.37%), while stacking showed the best generalization in testing with the minimum NRMSE (8.99%). In contrast, random forest and adaboost produced higher testing NRMSE values (>16%), indicating weaker predictive performance.

**Figure 4:**
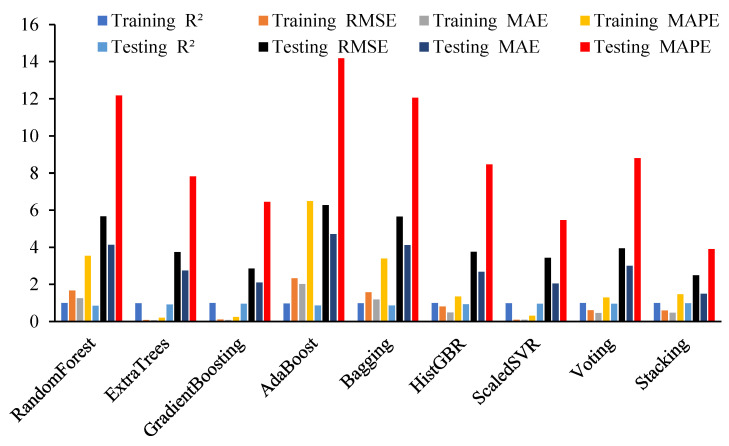
Comparative evaluation of ML models for predicting Mu.

SVR and ET also exhibited strong testing performance, with R^2^ values above 0.90, while tree-based models such as GB, RF, BG, and AB showed higher training accuracy but reduced testing performance, indicating moderate overfitting. The results indicate that omitting key geometric and material features impairs model generalization, while the SR consistently attains the highest predictive accuracy, followed by SVR and ET. Table 4 and Figure 4 compare ML model performance for Mu prediction, including L, h, and BFRP tensile strength, reporting R^2^, RMSE, MAE, and MAPE. RF achieved R^2^ = 0.990 (RMSE = 1.664, MAE = 1.255, MAPE = 3.536%) in training and 0.853 (RMSE = 5.668, MAE = 4.136, MAPE = 12.180%) in testing, while ET attained R^2^ = 0.981 (RMSE = 0.080, MAE = 0.065, MAPE = 0.200%) in training and 0.916 (RMSE = 3.746, MAE = 2.748, MAPE = 7.818%) in testing. NRMSE reflects the normalized prediction error of $M_{u}$, enabling scale-independent comparison among models. The stacking model achieved the lowest testing NRMSE (6.75%), whereas adaboost exhibited the highest (17.04%), indicating superior and weaker generalization performance, respectively.

**Figure 5:**
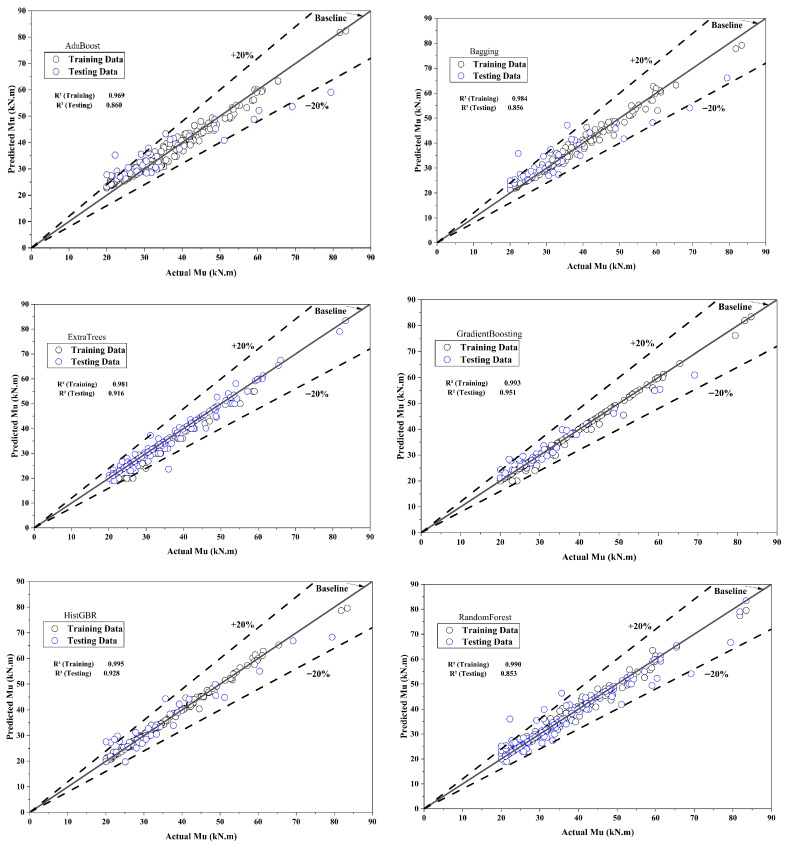

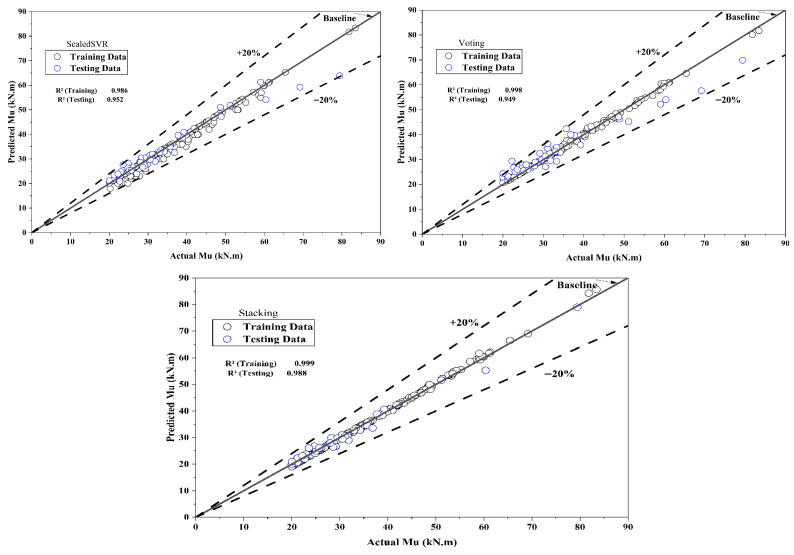
Comparison between observed and predicted Mu values for the training and testing datasets.

GB achieved R^2^ = 0.993 (RMSE = 0.103, MAE = 0.078, MAPE = 0.236%) in training and 0.951 (RMSE = 2.847, MAE = 2.104, MAPE = 6.452%) in testing, whereas AB recorded R^2^ = 0.969 (RMSE = 2.335, MAE = 2.015, MAPE = 6.492%) in training and 0.860 (RMSE = 6.278, MAE = 4.712, MAPE = 14.187%) in testing. BG achieved R^2^ = 0.984 (RMSE = 1.581, MAE = 1.190, MAPE = 3.392%) in training and 0.856 (RMSE = 5.654, MAE = 4.113, MAPE = 12.060%) in testing, while HGB reached R^2^ = 0.995 (RMSE = 0.801, MAE = 0.484, MAPE = 1.346%) in training and 0.928 (RMSE = 3.758, MAE = 2.684, MAPE = 8.461%) in testing. SVR attained R^2^ = 0.986 (RMSE = 0.098, MAE = 0.098, MAPE = 0.303%) in training and 0.952 (RMSE = 3.428, MAE = 2.041, MAPE = 5.464%) in testing, while VR achieved R^2^ = 0.998 (RMSE = 0.603, MAE = 0.456, MAPE = 1.295%) in training and 0.949 (RMSE = 3.941, MAE = 3.006, MAPE = 8.796%) in testing. The SR demonstrated the highest performance, achieving R^2^ = 0.999 (RMSE = 0.590, MAE = 0.472, MAPE = 1.465%) in training and R^2^ = 0.988 (RMSE = 2.487, MAE = 1.493, MAPE = 3.903%) in testing, reflecting superior predictive accuracy and generalization. Overall, including L, h and BFRP tensile strength improves model performance, with stacking showing the highest generalization and reliability. Figure 5 illustrates the comparison of observed and predicted Mu values for both training and testing datasets, highlighting the accuracy and generalization of the ML models. During model training, RMSE values were computed in the normalized feature space, and thus reflect errors in the scaled domain rather than the original $M_{u}$ range (20–91 kN·m). For physically interpretable and dimensionless evaluation, NRMSE (%) was calculated from the corresponding RMSE and the mean experimental ultimate moment using $\mathrm{NRMSE}=\frac{\mathrm{RMSE}}{{\bar{M}}_{u}}\times 100$.

**Figure 6:**
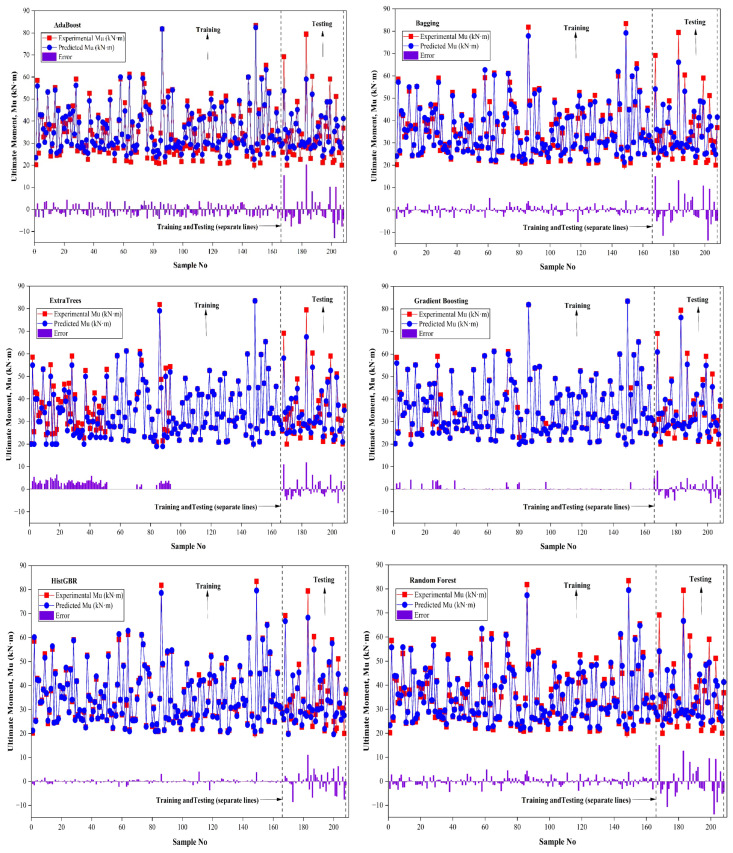

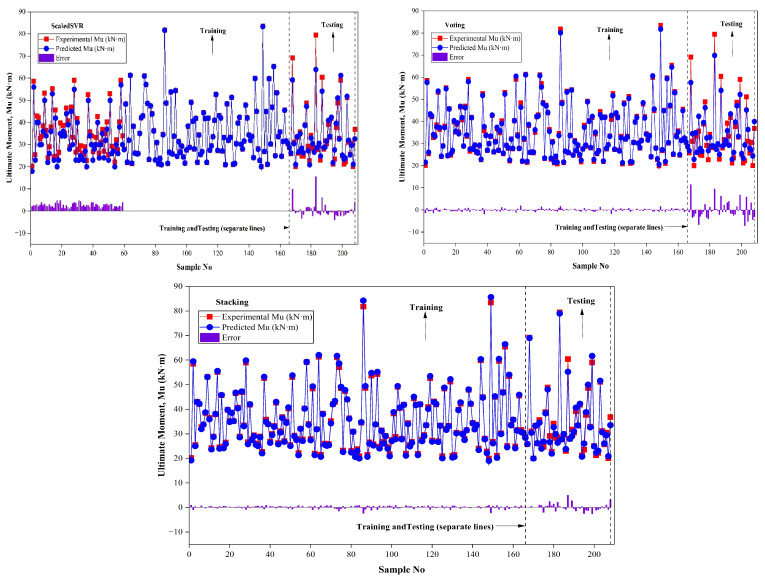
Predicted versus observed CS values with corresponding prediction errors for the training and testing datasets.

Figure 5 presents the comparison between predicted and actual Mu for training and testing datasets. The diagonal line represents the line of perfect agreement between predicted and actual responses, whereas the dashed lines correspond to ±20% prediction error limits. Ensemble learning models consistently outperform single learners, with boosting and stacking strategies, particularly gradient boosting, histogram-based gradient boosting, and stacking, achieving superior predictive accuracy and enhanced generalization performance. Figure 6 compares predicted and Mu values for training and testing datasets across different ML models, including the prediction errors. The vertical dashed line distinguishes the training and testing samples, while the lower panels show the residual errors for each model. Overall, the predicted values align closely with the experimental data, indicating satisfactory model performance. Single learners, such as SVM and AB, show higher test errors, whereas ensemble models like BG, RF, and boosting exhibit lower error dispersion and greater stability. The SR model exhibits the smallest residuals and the most consistent alignment with experimental values across both datasets, confirming its superior accuracy and generalization capability.

All input features were normalized, and outliers were removed using the Z-score and IQR methods. Hyperparameter optimization, a computationally intensive iterative process, was then applied to identify the best model configurations. After these steps, the stacking model achieved R^2^ = 0.988 on the test set, with 10-fold cross-validation confirming stability and Taylor diagrams indicating high correlation and low RMSE. These results demonstrate that rigorous preprocessing and hyperparameter tuning are essential for genuine predictive capability. For future work, study-wise grouped validation is recommended to assess inter-study generalization.

**Figure 7:**
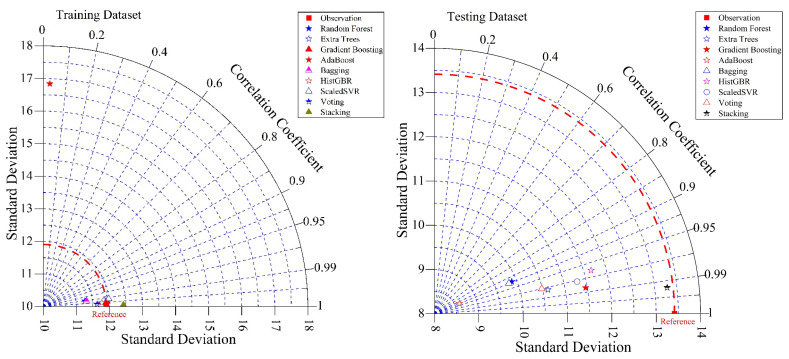
Taylor diagram of ML model performance for training and testing datasets.

**Figure 8:**
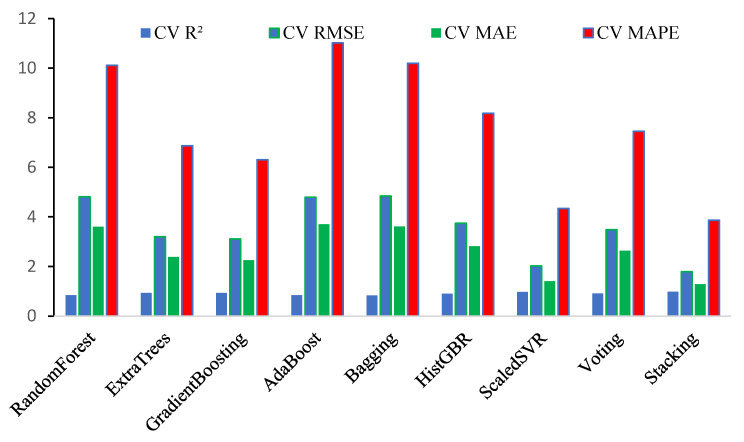
Comparative assessment of ML models using 10-fold cross-validation for predicting Mu.

Figure 7 presents Taylor diagrams summarizing model performance for the training and testing datasets, showing correlation, standard deviation, and centered RMSE relative to the reference observations. Models nearer the reference point in both datasets exhibit higher correlation and closer agreement with the observed ultimate moment values. For the training dataset, ensemble models, particularly SR, exhibit correlations and standard deviations closely matching the observations, reflecting effective capture of data variability. In the testing dataset, SR, GB, and HGB lie closest to the reference, indicating superior generalization and lower error. Single learners exhibit greater deviations and lower correlations, whereas the Taylor diagrams confirm that ensemble and meta-learning models provide the most reliable and accurate predictions of ultimate moment capacity. Table 5 and Figure 8 summarize the results of 10-fold cross-validation for the evaluated ML models in predicting the ultimate moment capacity of BFRP-reinforced concrete beams. Among the individual models, GB and ET exhibited strong predictive performance, with R^2^ values exceeding 0.92 and relatively low error metrics, indicating an effective ability to capture nonlinear relationships. SVR achieved the highest predictive accuracy among the single models, with an R^2^ of 0.971 and the lowest RMSE, MAE, and MAPE values, indicating superior prediction performance. Simple ensemble methods, including RF, AB, and BG, exhibited moderate predictive performance, characterized by lower R^2^ values and comparatively higher error metrics. As indicated in Table 5, the 10-fold cross-validation NRMSE values demonstrate that stacking (4.84%) and SVR (5.49%) achieved the lowest normalized errors, reflecting superior robustness, whereas bagging (13.13%), randomforest (13.04%), and adaboost (13.00%) exhibited comparatively higher NRMSE values, indicating weaker generalization performance.

Advanced ensemble strategies, such as voting and stacking, further enhanced predictive accuracy, with the stacking model achieving the best overall performance (R^2^ = 0.977, RMSE = 1.784 kN·m, and MAPE = 3.87%), highlighting the effectiveness of integrating multiple base learners. Overall, the results indicate that meta-ensemble and kernel-based approaches provide the most reliable predictions of Mu, whereas simpler ensemble methods yield reasonable but comparatively less precise estimates.

**Figure 9:**
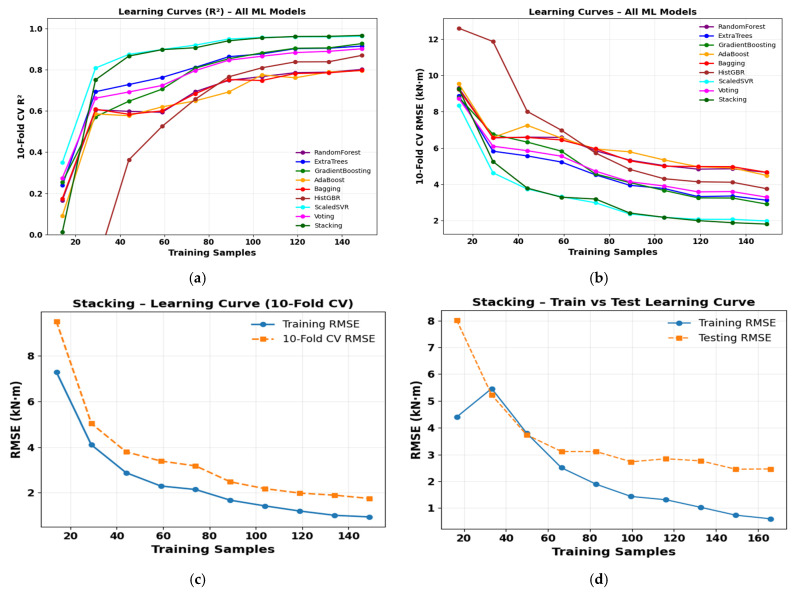
Learning curves of ML models.

Figure 9 illustrates the learning curves of the evaluated ML models, showing their predictive performance as a function of training sample size. Figure 9a demonstrates that the cross-validated R^2^ increases with sample size and subsequently converges, indicating stabilized predictive performance, while ensemble models, particularly the stacking approach, consistently exhibit superior accuracy. Figure 9b–d show that increasing data reduces RMSE and narrows the training–validation gap for the stacking model, indicating improved accuracy, limited overfitting, and strong generalization, evidenced by a stable and small training–testing RMSE difference at larger sample sizes. Despite achieving high predictive performance using hyperparameter-optimized ensembles with 80/20 training–testing and 10-fold cross-validation, the moderate dataset 220 specimens may constrain generalizability. Future work will expand the dataset and employ external or leave-one-study-out validation to strengthen model robustness and reliability.

The dataset of 208 curated specimens was rigorously validated to ensure representativeness and robustness, with outliers removed using Z-score and IQR analyses and distributional characteristics confirmed via the Kolmogorov–Smirnov test. All models were hyperparameter-optimized via grid search, with the stacking ensemble achieving R^2^ = 0.999 for training, R^2^ = 0.988 for testing, and an average 10-fold cross-validation R^2^ = 0.977 (RMSE = 1.784, MAE = 1.288, MAPE = 3.865%), demonstrating high predictive accuracy. Error analysis, residual distributions, Taylor diagrams, and learning curves collectively confirm minimal prediction errors, strong correlation with observed data, limited overfitting, and effective generalization, supporting the reliability of the proposed framework.

Table 6 summarizes the predictive accuracy and robustness of the implemented ML models for estimating the Mu using 10 × 10 repeated K-fold cross-validation. The mean and standard deviation values of R^2^, RMSE, and MAE reflect both model accuracy and performance variability across different data partitions. Among the evaluated models, the stacking regressor (SR) achieved the highest accuracy (R^2^ = 0.971) and the lowest prediction errors (RMSE = 1.877 and MAE = 1.400), indicating superior predictive capability. Moreover, the relatively low standard deviations associated with SR suggest enhanced stability and generalization performance. These findings demonstrate the effectiveness of the stacking ensemble approach in modeling the nonlinear flexural response of FRP-reinforced concrete beams. The stacking regressor (SR) produced a mean flexural capacity $\left(M_{u}\right)$ predictions of 23.28, 23.25, 29.89, 36.75, and 25.12 kN·m for the first five samples, with corresponding 95% prediction intervals of [21.89–30.24], [21.74–28.96], [23.67–33.17], [24.17–39.99], and [22.03–29.98] kN·m, respectively. The interval widths, varying between 7.22 and 15.82 kN·m, indicate moderate predictive uncertainty and reflect stable generalization performance across different input configurations. The relatively wider interval associated with the fourth sample implies increased variance in prediction, potentially arising from nonlinear interactions among input variables.

Table 7 summarizes the results of the permutation-based randomization test conducted to evaluate model robustness. Upon shuffling the target variable, all models exhibited negative R^2^ values, ranging from −0.231 ± 0.056 (RF) to −0.710 ± 0.157 (stacking regressor). The stacking model showed the strongest degradation (R^2^ = −0.710 ± 0.157), followed by SVR (−0.670 ± 0.155) and GB (−0.622 ± 0.157), indicating complete loss of predictive capability under noise conditions. The consistently negative R^2^ values confirm that the models are unable to extract artificial structure from randomized data, thereby demonstrating resistance to spurious learning and validating the reliability of the original predictive performance.

**Figure 10:**
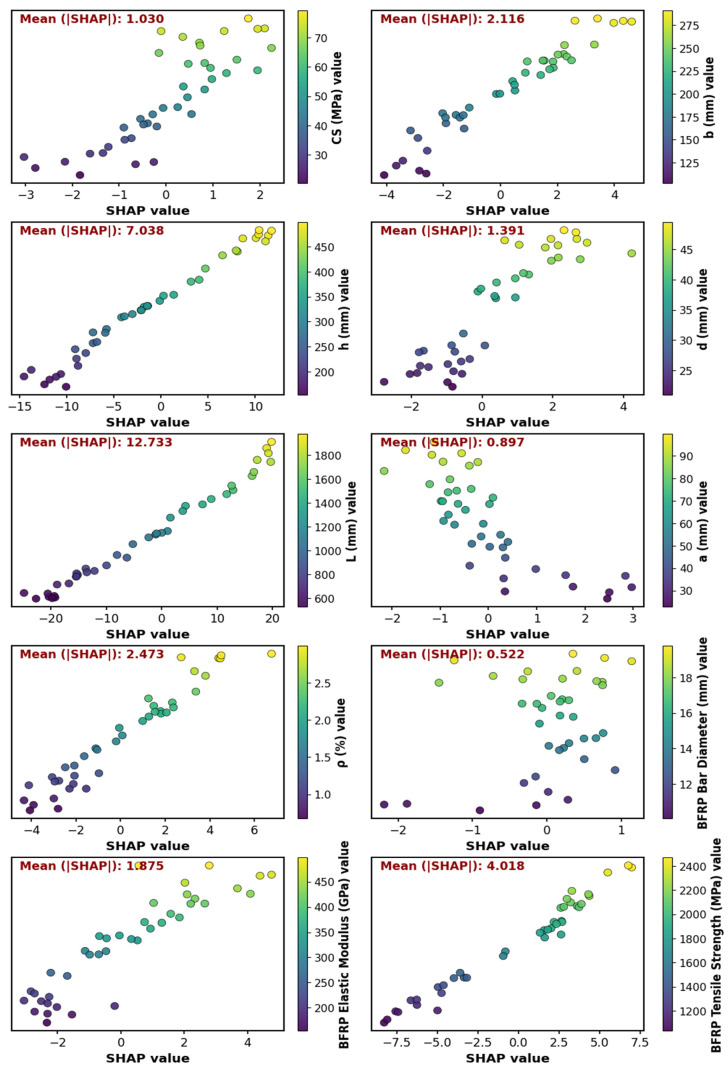
SHAP dependence plots of input features.

Figure 10 presents SHAP dependence plots for the input variables, quantitatively illustrating both the direction and magnitude of their contributions to the model predictions. The beam length L exhibits the highest influence (mean SHAP = 12.733), followed by the section height h (7.038), underscoring the predominant role of geometric parameters. The material properties of BFRP also exhibit a notable influence on the predictions, as evidenced by the tensile strength and elastic modulus, with mean SHAP values of 4.018 and 1.875, respectively. The BFRP ratio ρ and beam width b show moderate influence (mean SHAP = 2.473 and 2.116), while concrete compressive strength and effective depth d exhibit lower but non-negligible contributions (mean SHAP = 1.030 and 1.391). In contrast, the bar diameter and cover-related parameters exhibit the least influence, with mean SHAP values below 1.0, indicating a minimal effect on the model predictions.

**Figure 11:**
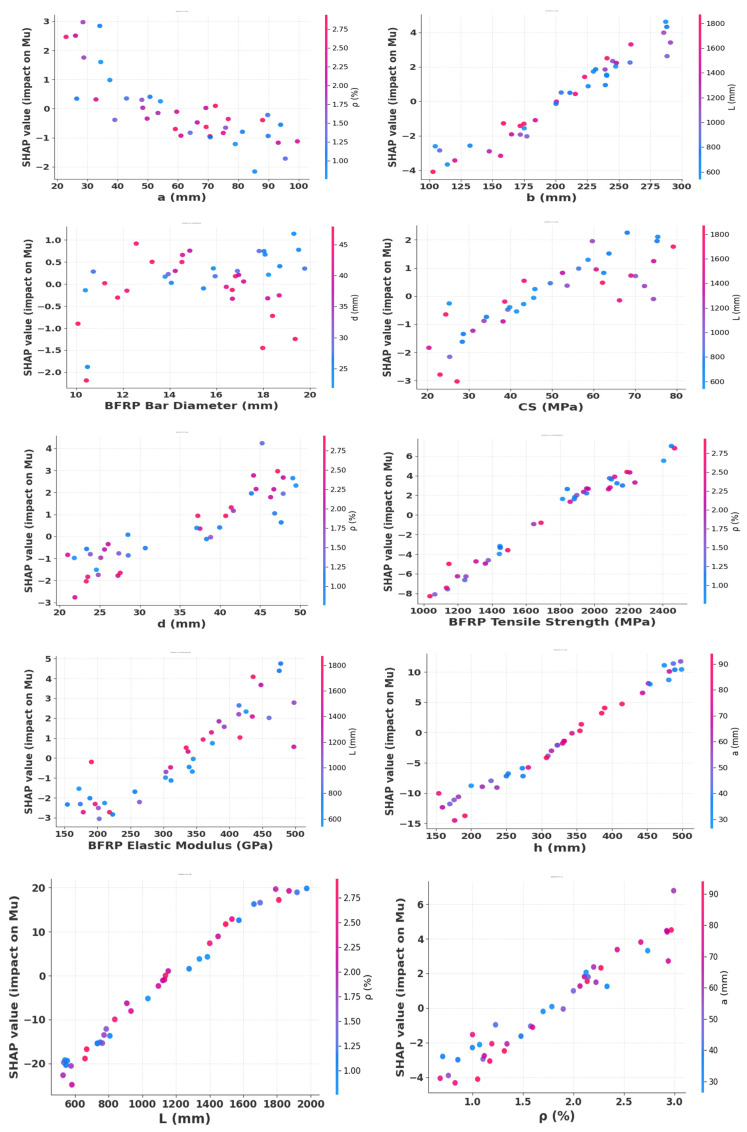
SHAP interaction plot showing interaction effects between input variables.

Figure 11 presents SHAP interaction plots that systematically quantify both the individual contributions and coupled effects of key input variables on the predicted Mu. Geometric parameters, particularly the beam length L and section height h, exhibit the strongest positive influence on the predicted response. The BFRP material properties, notably tensile strength and elastic modulus, exert a significant positive influence on the predictions, whereas the bar diameter exhibits a comparatively secondary effect. The interaction plots further demonstrate strong nonlinear coupling between beam length and reinforcement ratio, and between reinforcement ratio and shear span, leading to amplified SHAP contributions at higher parameter levels. Overall, these findings confirm that Mu is governed by complex nonlinear interactions among geometric, material, and reinforcement parameters, rather than by independent effects alone.

**Figure 12:**
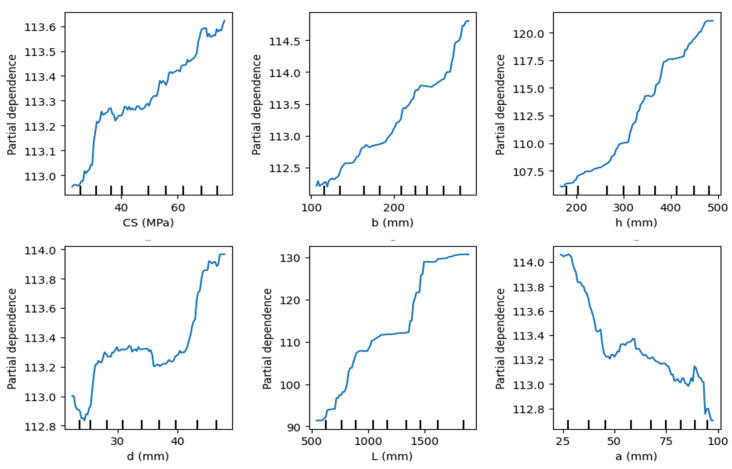

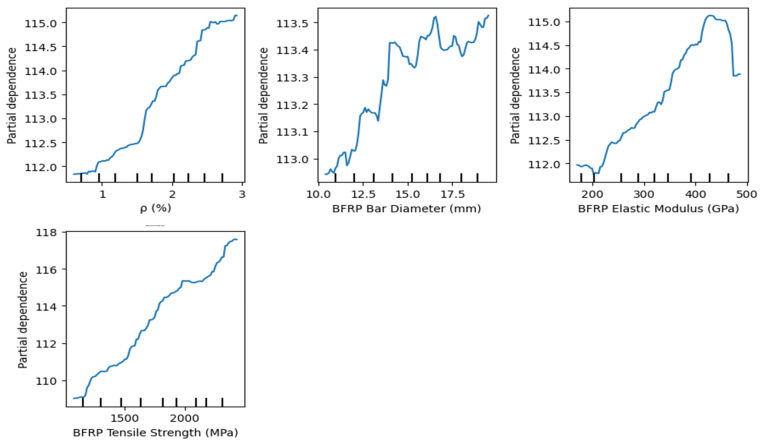
Partial dependence plots of input features.

Figure 12 presents partial dependence plots (PDPs) showing the marginal effects of input variables on the predicted bending Mu. Concrete compressive strength (CS) and beam width (b) demonstrate limited yet positive contributions, resulting in an approximate increase of 0.5–1.5 units in Mu over their variation ranges. Beam height h and span length L exhibit the strongest nonlinear effects, with the predicted capacity increasing by approximately 12–25 units, underscoring their dominant structural role. The effective depth d and reinforcement ratio ρ exhibit threshold-type behavior, characterized by initial increases in capacity followed by saturation. In contrast, the shear span a exhibits a negative marginal effect, reducing Mu by approximately 1–1.5 units as its value increases. The BFRP material properties, particularly tensile strength, consistently enhance the predicted bending moment capacity. Overall, the PDPs confirm nonlinear yet physically interpretable relationships, supporting the reliability of the ML model.

**Figure 13:**
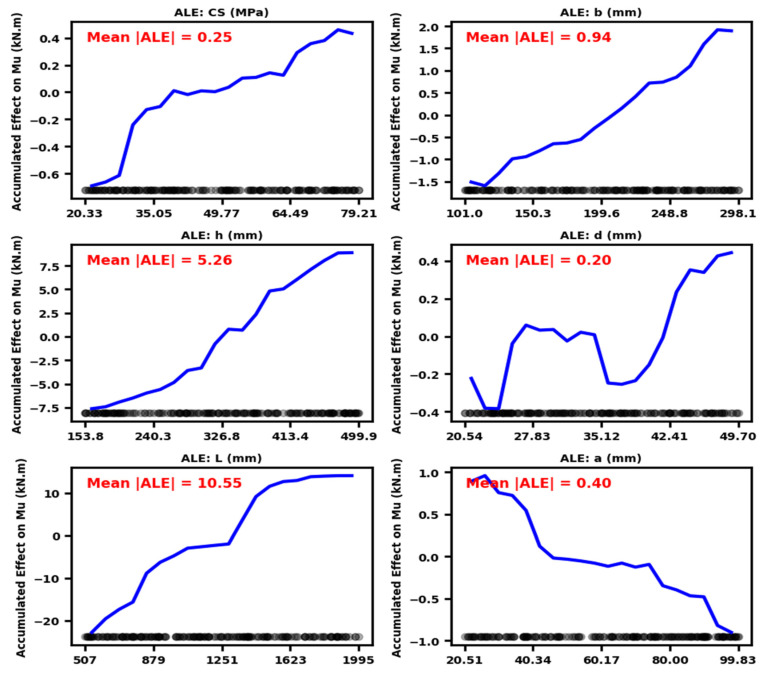

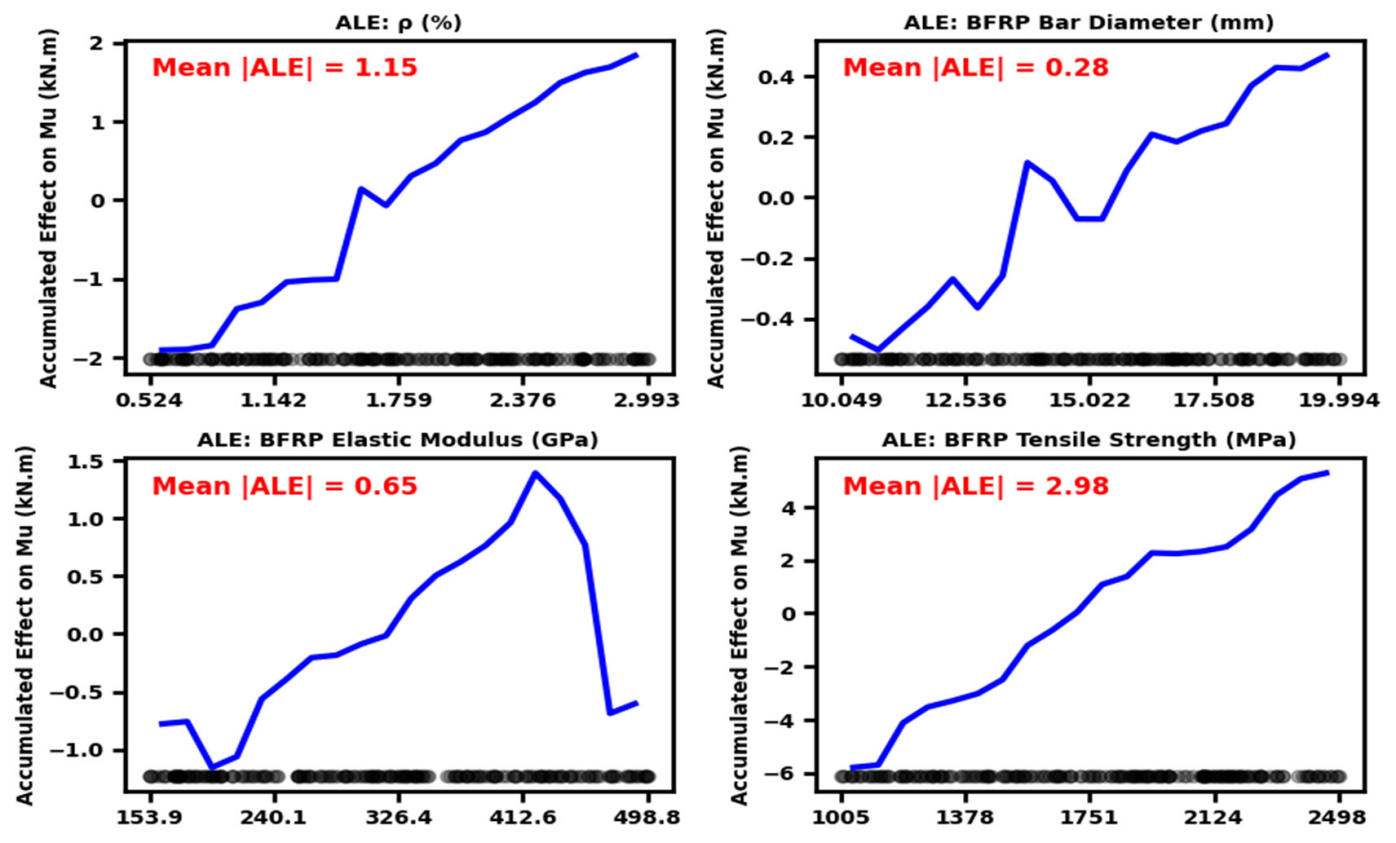
ALE plots showing the local effects of input variables on the model response.

Figure 13 depicts the accumulated local effects (ALE) of the input variables on the predicted $M_{u}$. The mean absolute ALE values provide a concise and objective quantitative measure of the relative contribution of each parameter to the model response. The analysis demonstrates that geometric parameters predominantly govern the model response, with beam span length L exerting the greatest influence (Mean |ALE|=10.55 kN·m), followed by overall beam depth h (5.26 = kN·m), highlighting the critical role of global geometry in flexural capacity. Among material-related variables, BFRP tensile strength exhibits a substantial effect (Mean |ALE|=2.98 kN·m), whereas the reinforcement ratio ρ contributes moderately to enhancing flexural performance (1.15 kN·m). Beam width b also exhibits a noticeable influence on the model response (0.94 kN·m). In contrast, parameters with comparatively lower impact include the BFRP elastic modulus (0.65 kN·m), shear span a (0.40 kN·m), bar diameter (0.28 kN·m), concrete compressive strength (0.25 kN·m), and effective depth d (0.20 kN·m). Overall, the ALE analysis verifies that the model reflects physically consistent behavior, with global geometry governing the response, followed by BFRP mechanical properties, while concrete strength and secondary geometric parameters have a minor influence.

**Figure 14:**
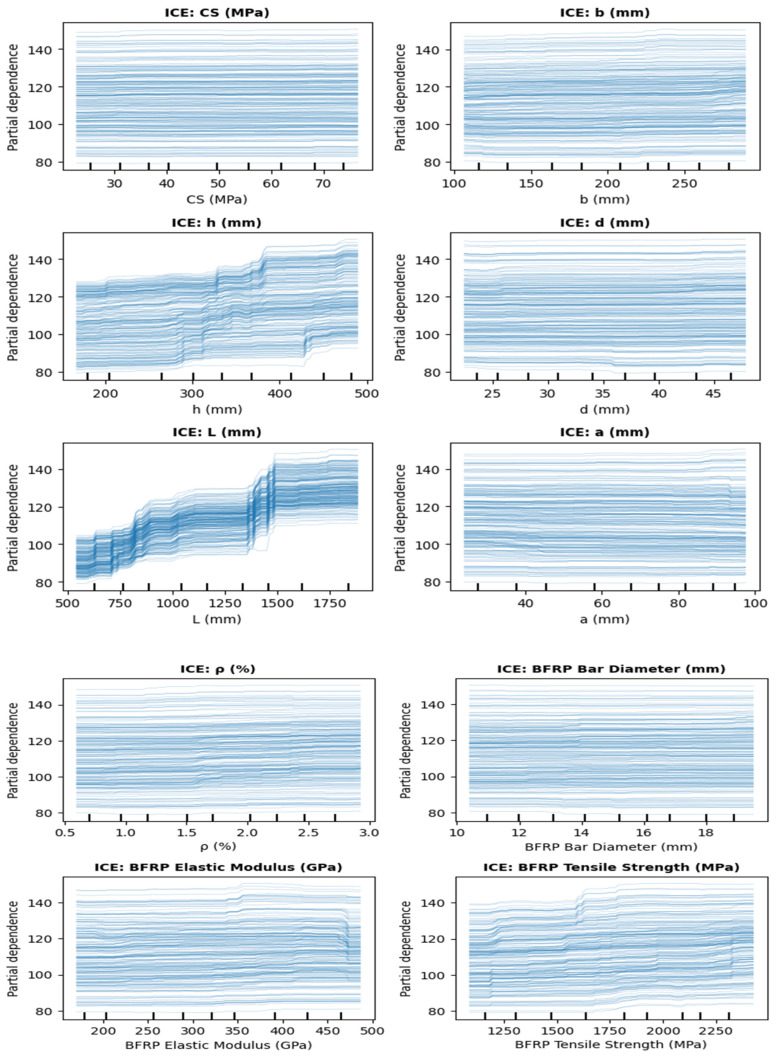
Individual conditional expectation (ICE) plots showing the effect of input features on the predicted response.

Figure 14 presents the individual conditional expectation (ICE) plots, illustrating the marginal influence of each input variable on the predicted flexural response. The predominantly parallel and gently sloped curves associated with concrete compressive strength and effective depth indicate a weak, approximately linear influence on the model response. In contrast, beam depth h and span length L show pronounced upward trends with greater dispersion, indicating dominant and nonlinear effects on flexural capacity, whereas beam width b exhibits a moderate yet consistent positive influence reflecting its secondary geometric role. Among material parameters, BFRP tensile strength exhibits a clear positive influence with moderate variability, whereas the elastic modulus shows a weaker effect, indicating reduced sensitivity at higher stiffness levels. Reinforcement ratio ρ and bar diameter display limited slopes, indicating incremental rather than governing contributions to the predicted response. The ICE plots confirm that the model captures physically interpretable relationships, with global geometric parameters governing the response and material properties exerting a secondary influence.

**Figure 15:**
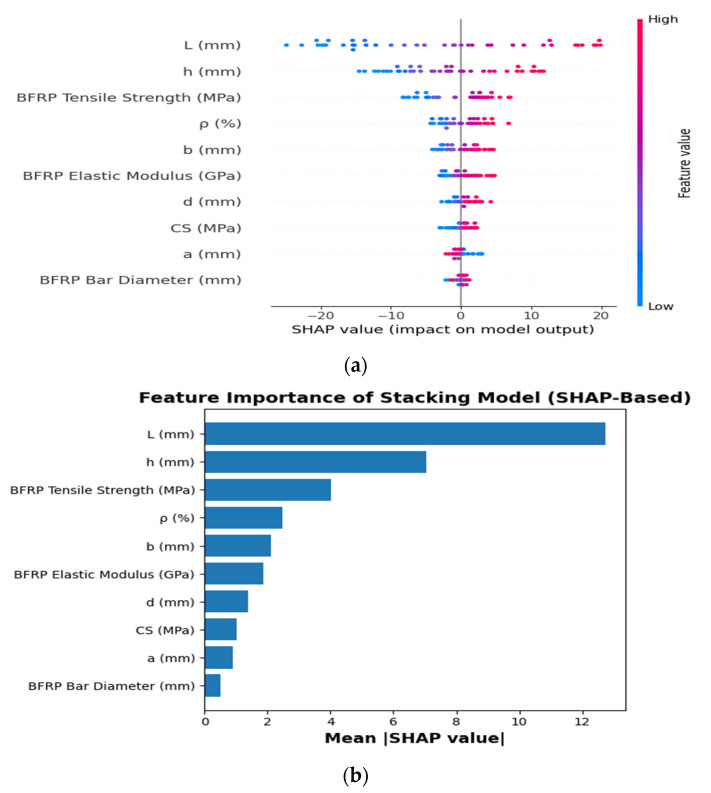
SHAP summary plot of input features based on the Stacking model.

Figure 15 presents the SHAP-based interpretation of the stacking model, elucidating the relative contributions of the input features to the predicted flexural response. The results indicate that beam span length L and depth h are the most influential variables, followed by BFRP tensile strength, whereas the reinforcement ratio and beam width exhibit moderate effects. Secondary geometric parameters and concrete compressive strength exhibit comparatively minor contributions. Overall, the SHAP analysis confirms that the model captures physically consistent and interpretable relationships.

**Figure 16:**
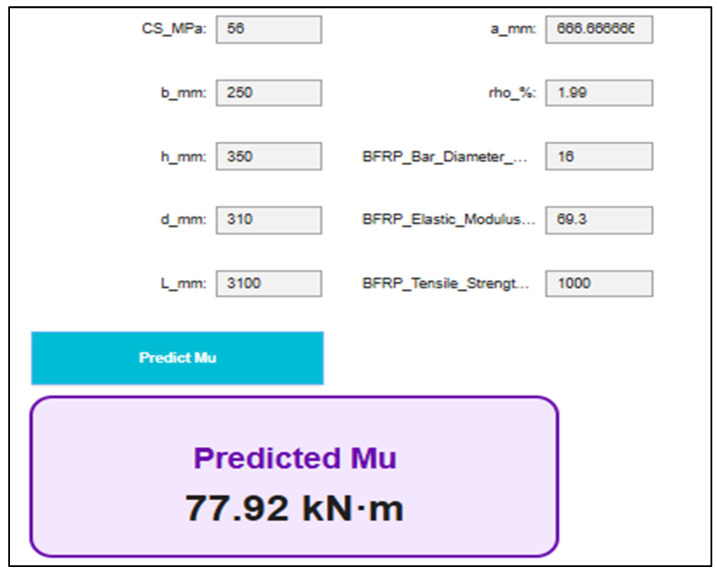
GUI of the best ML model (SR) for Fc prediction.

The observed low influence of concrete compressive strength (CS) on flexural capacity (Mu) reflects the narrow strength range considered (30–65 MPa) and may not generalize to beams with wider strength variations. In classical reinforced concrete theory, CS affects Mu via stress block parameters; thus, the results should be interpreted in the context of the dataset’s constraints. An interactive graphical user interface (GUI) was developed for predicting the ultimate moment capacity $M_{u}$ using the proposed stacking regressor model, as illustrated in Figure 16. The interface incorporates the relevant geometric and material input parameters of BFRP-reinforced concrete beams, including beam dimensions, reinforcement characteristics, and BFRP mechanical properties. All input variables are processed using the same robust and min–max scaling procedures employed during model training, thereby ensuring consistency between the training and inference stages and enhancing the reliability of the predicted responses. The GUI enables users to dynamically modify input values and obtain real-time $M_{u}$ predictions, thereby facilitating rapid parametric studies and enhancing the practical applicability of the ML model in engineering design and assessment.

The validity of the proposed ML framework is explicitly confined to the parametric domain represented by the experimental database employed for model development and validation. The predictive algorithm and associated graphical user interface are therefore applicable exclusively within the calibrated ranges of concrete compressive strength (30–65 MPa), beam span length (2000–3100 mm), BFRP reinforcement ratio (0.003–2%), and BFRP elastic modulus (42.8–69.3 GPa). Any prediction generated beyond these bounds constitutes statistical extrapolation outside the training data manifold and may compromise physical reliability and predictive stability. In addition to numerical parameter constraints, the structural scope of the model is limited to BFRP-reinforced concrete beams subjected to static monotonic flexural loading under laboratory-controlled conditions. The framework does not incorporate cyclic or fatigue loading effects, long-term time-dependent phenomena (e.g., creep and shrinkage), environmental degradation, elevated temperature exposure, serviceability performance, or hybrid reinforcement configurations. Consequently, the model should be interpreted as a data-driven predictive tool operating within a rigorously defined applicability domain, rather than a universally generalizable design formulation.

### 3.1. Performance Validation of the Extra Trees Predictive Model

This section evaluates the predictive performance of the stacking regressor model for estimating the ultimate moment capacity of BFRP-reinforced concrete beams, with comparisons to prior studies (Table 8). An 80/20 train–test split with 10-fold cross-validation was adopted. Model performance was evaluated using MAE, RMSE, R^2^, and MAPE, while PDP, SHAP, ALE, and ICE analyses confirmed the reliability and interpretability of the SR model. Table 6 compares the proposed stacking regressor with prior studies, showing that it achieves superior accuracy (training R^2^ = 0.999, testing R^2^ = 0.988) and minimal errors (RMSE = 0.590–2.487, MAE = 0.472–1.493%, MAPE = 1.465–3.903%) on a dataset of 220 samples. In comparison, previous models including ANN, GEP, SVM, and regression methods exhibit lower predictive performance across varied dataset sizes and train–test splits. These results highlight the robustness and generalization capability of the stacking regressor for reliable Mu prediction.

The flexural capacities were evaluated in accordance with ACI 440.1R-15 provisions, based on rigorous application of strain compatibility and internal force equilibrium principles. The predicted nominal moment capacity, $M_{u,ACI}$, was systematically compared with the experimentally measured ultimate moment, $M_{u,exp}$. Model performance was assessed using *R*^2^, *RMSE*, *MAE*, and *MAPE*, as defined below.

$$R^{2}=1-\frac{\sum (M_{u,exp}-M_{u,ACI}{)}^{2}}{\sum (M_{u,exp}-{\bar{M}}_{u,exp}{)}^{2}}$$

$$RMSE=\sqrt{\frac{1}{n}\sum (M_{u,exp}-M_{u,ACI}{)}^{2}}$$

$$MAE=\frac{1}{n}\sum \left|M_{u,exp}-M_{u,ACI}\right|$$

$$MAPE=\frac{100}{n}\sum \left|\frac{M_{u,exp}-M_{u,ACI}}{M_{u,exp}}\right|$$

where n denotes the number of specimens and ${\bar{M}}_{u,exp}$ represents the mean experimental ultimate capacity. For the ACI model, $R^{2}=0.352$ indicates limited predictive correlation with the experimental data, accompanied by relatively large discrepancies $\left(RMSE=11.043,MAE=9.881,MAPE=28.98\%\right)$, reflecting an average deviation of approximately 29%. In contrast, the stacking ensemble model demonstrated substantially enhanced predictive accuracy $\left(R^{2}=0.988,RMSE=2.487,MAE=1.493,MAPE=3.903\%\right)$, indicating superior generalization capability relative to the conventional design provisions. In addition to conventional error metrics, the predictive bias and dispersion were evaluated using the mean prediction ratio (Mean λ) and coefficient of variation (COV). The ACI 440.1R-15 model exhibited a pronounced underestimation bias (Mean λ ≈ 0.71) with considerable variability (COV ≈ 0.30), whereas the stacking ensemble model demonstrated nearly unbiased predictions (Mean λ ≈ 0.99) and substantially reduced dispersion (COV ≈ 0.04). These results confirm not only improved accuracy but also enhanced reliability and statistical stability of the proposed machine learning framework compared to conventional design provisions.

### 3.2. Mechanical Interpretation of Key Parameter Interactions

Although advanced interpretability frameworks yield quantitative evaluations of feature significance and interaction effects, their outcomes require rigorous validation within the principles of structural mechanics to ensure consistency with fundamental engineering behavior [94]. This validation ensures that the inferred relationships remain physically consistent with classical flexural theory and fundamental mechanics. This section interprets the key data-driven interactions within the framework of equilibrium, strain compatibility, and sectional moment resistance in BFRP-reinforced concrete beams. Figure 11 reveals a pronounced nonlinear coupling between span length (L) and reinforcement ratio (ρ), highlighting their interdependent effect on the flexural performance of the section. From a structural mechanics standpoint, the bending moment demand in a simply supported beam under uniformly distributed load scales quadratically with span length, expressed as $M_{\max}=\frac{wL^{2}}{8}$. This quadratic dependence intensifies the influence of reinforcement ratio on flexural performance, providing a mechanistic explanation for the observed nonlinear interaction [95]. The quadratic increase in flexural demand with L contrasts with the nominal moment capacity of a BFRP-reinforced section, $M_{n}=T\cdot z=A_{f}f_{f}\cdot z$, where $A_{f}\;$(∝ ρ) is the reinforcement area, $f_{f}$ the tensile stress, and z the lever arm, are determined by strain compatibility and neutral axis depth. Since BFRP reinforcement is linearly elastic up to rupture, ρ controls both tensile capacity and the resulting strain distribution, including neutral axis depth. Although bending demand scales with $L^{2}$, strain compatibility and compression limits constrain proportional increases in $M_{u}$, yielding a nonlinear, configuration-dependent response. The nonlinear L–ρ interaction revealed by SHAP reflects the coupling between span-driven moment demand and reinforcement-controlled tensile resistance, aligning with classical flexural equilibrium and physically consistent behavior.

Explainable AI confirms that flexural performance is primarily governed by the classical interplay between span-induced demand (∝ L^2^) and depth-dependent resistance (∝ h^2^). ALE analysis (Figure 13) identifies span length (L) and beam depth (h) as the key predictors of ultimate moment capacity, consistent with classical flexural theory [96]. The nominal moment capacity scales with $M_{n}\propto bd^{2}$ while bending demand scales with $L^{2}$; consequently, the span-to-depth ratio (L/d) serves as the principal parameter governing stiffness, curvature, strain distribution, and the failure mode of the section [97]. Beams with higher L/d ratios exhibit increased curvature and tensile strains, while greater depth enhances the lever arm and compression capacity, improving flexural resistance. The agreement among SHAP, PDP, ALE, and ICE analyses confirms that the stacking model captures physically consistent relationships. Global geometry (L and h) chiefly dictates flexural capacity, with reinforcement ratio and BFRP strength exerting secondary, configuration-dependent effects, and concrete strength and bar diameter playing a minor role under flexure-controlled behavior. The explainable ML framework captures higher-order nonlinear interactions consistent with equilibrium, strain compatibility, and moment resistance, confirming the model’s physical validity and reliability. To quantitatively substantiate the mechanics-aligned interpretability claim, the SHAP-derived feature trends were explicitly compared with classical flexural capacity formulations. According to ACI 440.1R, the nominal flexural capacity of FRP-reinforced concrete beams is expressed as $M_{n}=A_{f}f_{f}(d-a/2)$, where the capacity scales approximately linearly with effective depth d and proportionally with reinforcement area $A_{f}$, while nonlinear behavior arises from strain compatibility constraints governing FRP stress development. The SHAP dependence plots reveal (i) a strong monotonic and near-linear increase in SHAP values with both effective depth and total depth, consistent with $M_{n}\propto d$; (ii) a nonlinear but positive sensitivity to reinforcement ratio, reflecting compatibility-controlled stress limits; and (iii) monotonic enhancement with FRP tensile strength and modulus, consistent with increased stress transfer capacity. Furthermore, the pronounced SHAP contribution of span length reflects structural scaling of bending moment demand ($M\sim L^{2}$). These quantitative correspondences confirm that the model’s learned relationships are physically grounded in equilibrium-based flexural mechanics rather than purely data-driven correlations.

## 4. Conclusions

This study proposes an interpretable ML-based framework, supported by ensemble modeling and explainable AI techniques, for predicting the Mu of BFRP-reinforced concrete beams using a comprehensive experimental database. The framework provides a systematic alternative to traditional design approaches while enhancing interpretability. Although the stacking ensemble exhibits high accuracy, the moderate dataset size and experimental variability may limit generalizability. The study is limited to static monotonic flexural behavior of BFRP-reinforced beams under laboratory conditions, excluding time-dependent, durability, serviceability, and hybrid reinforcement effects. Future research should expand datasets, include long-term behavior, and develop physics-informed or code-oriented ML models to improve generalizability and applicability. The principal conclusions of the present study can be summarized.

1. Among the evaluated models, ensemble learning approaches demonstrated consistently superior accuracy and robustness compared with individual learners.
2. The stacking regressor (SR) demonstrated superior predictive capability and generalization performance, achieving an excellent agreement during training (R^2^ = 0.999, RMSE = 0.590 kN·m) and maintaining high accuracy in testing (R^2^ = 0.988, RMSE = 2.487 kN·m), and the best performance under 10-fold cross-validation (R^2^ =0.977, RMSE = 1.784 kN·m).
3. The inclusion of beam span length L, depth h, and BFRP tensile strength markedly enhances model performance. For the SR, testing accuracy increased from R^2^ = 0.932 to 0.988 (≈6% improvement), while RMSE and MAPE were reduced by approximately 25%, confirming the critical contribution of these parameters to predictive accuracy and generalization.
4. Among the evaluated models, adaboost exhibited the weakest performance, achieving a testing accuracy of R^2^ = 0.86 with high prediction errors (RMSE = 6.28 kN·m and MAPE = 14.19%), indicating limited generalization and reduced capability in capturing the nonlinear flexural behavior of BFRP-reinforced concrete beams.
5. Explainable AI analyses (SHAP, PDP, ALE, and ICE) indicate that span length and section depth dominate flexural capacity, with secondary influence from BFRP tensile strength and minor contributions from concrete strength and other geometric parameters.
6. The agreement among multiple explainability techniques confirms the physical plausibility of the learned relationships.
7. The integration of high predictive accuracy with robust interpretability enables reliable engineering assessment and supports informed, evidence-based decision-making.
8. The incorporation of a rigorous mechanics-based interpretation enhances the scholarly robustness of the study by establishing that the explainable ML outcomes are theoretically grounded in classical flexural mechanics and consistent with fundamental demand–capacity relationships governing reinforced concrete beam behavior.
9. Overall, the proposed machine learning framework constitutes a reliable, transparent, and practically applicable alternative to conventional design equations for BFRP-reinforced concrete beams.

## Figures and Tables

**Table 1:** Descriptive statistics, normality (Kolmogorov–Smirnov, α = 0.05), and multicollinearity (VIF) of input features.

Parameter	Symbol	Unit	Mean	Std	Min	Max	VIF	KS Statistic	*p*-Value
Compressive Strength	CS	MPa	43.643	9.561	30	65	1.024	0.121	0.003
Beam Width	b	mm	215.682	26.552	180	250	1.075	0.227	0.000
Beam Depth	h	mm	303.818	39.227	230	350	92.218	0.240	0.000
Effective Depth	d	mm	258.995	39.090	190	310	91.566	0.212	0.000
Span Length	L	mm	2448.636	417.590	2000	3100	215.177	0.251	0.000
Shear Span	a	mm	820.455	145.247	666.667	1100	222.527	0.217	0.000
Reinforcement Ratio	ρ	%	1.315	0.500	0.003	2	1.660	0.102	0.019
BFRP Bar Diameter	dBFRP	mm	12.700	2.607	8.000	16	1.019	0.252	0.000
BFRP Elastic Modulus	EBFRP	GPa	50.390	4.998	42.800	69.300	1.221	0.195	0.000
BFRP Tensile Strength	Fu, BFRP	MPa	1116.069	149.457	900.100	1760	1.523	0.084	0.084
Ultimate Moment	Mu	kN·m	37.065	14.081	20	91.030			

**Table 2:** Hyperparameter configuration of ML models used for Mu prediction.

Model	Hyperparameters
Random Forest (RF)	n_estimators = 250, max_depth = 6, random_state = 42
Extra Trees (ET)	n_estimators = 250, max_depth = 7, random_state = 42
Gradient Boosting (GB)	n_estimators = 260, learning_rate = 0.1, max_depth = 3, subsample = 1.0, random_state = 42
AdaBoost (AB)	Base learner: Decision Tree (max_depth = 4), n_estimators = 300, learning_rate = 1.0, loss = linear, random_state = 42
Bagging (BG)	Base learner: Decision Tree, n_estimators = 245, max_samples = 1.0, max_features = 1.0, random_state = 42
Histogram-based Gradient Boosting (HGB)	max_iter = 310, learning_rate = 0.1, max_depth = 6, random_state = 42
Support Vector Regressor (SVR)	Kernel = RBF, C = 89, gamma = scale, epsilon = 0.1, preprocessing: StandardScaler
Voting Regressor (VR)	RF (n_estimators = 200) + ET (n_estimators = 200) + GB (n_estimators = 200), equal weights
Stacking Regressor (SR)	Base learners: RF (n_estimators = 200), GB (n_estimators = 200), SVR (C = 100); Meta-learner: Linear Regression

**Table 3:** Training and testing performance of ML models for Mu prediction without L, h and BFRP tensile strength.

Model	Training					Testing				
	R^2^	RMSE	MAE	MAPE	NRMSE	R^2^	RMSE	MAE	MAPE	NRMSE
Scaled SVR	0.975	0.807	0.173	0.40	2.19	0.900	3.535	3.130	6.123	9.59
ExtraTrees	0.954	0.904	0.965	1.126	2.45	0.9052	4.139	2.987	7.675	11.23
Stacking	0.9934	0.765	0.678	1.564	2.08	0.932	3.313	2.335	5.25	8.99
GradientBoosting	0.981	0.163	0.119	0.37	0.44	0.8315	5.517	2.796	6.72	14.97
Voting	0.990	0.695	0.487	1.31	1.89	0.901	4.230	2.781	8.890	11.48
HistGBR	0.988	1.563	0.964	2.58	4.24	0.8337	5.480	3.031	9.145	14.87
RandomForest	0.982	1.885	1.305	3.886	5.12	0.843	6.567	4.567	12.456	17.82
Bagging	0.981	1.981	1.356	3.57	5.38	0.843	6.037	4.253	12.567	16.39
AdaBoost	0.9557	2.987	2.458	8.27	8.11	0.8428	6.329	4.787	14.57	17.17

**Table 4:** Comparative performance of ML models in predicting Mu.

Model	Training					Testing				
	R^2^	RMSE	MAE	MAPE	NRMSE	R^2^	RMSE	MAE	MAPE	NRMSE
RandomForest	0.990	1.664	1.255	3.536	4.52	0.853	5.668	4.136	12.180	15.38
ExtraTrees	0.981	0.080	0.065	0.200	0.22	0.916	3.746	2.748	7.818	10.17
GradientBoosting	0.993	0.103	0.078	0.236	0.28	0.951	2.847	2.104	6.452	7.73
AdaBoost	0.969	2.335	2.015	6.492	6.34	0.860	6.278	4.712	14.187	17.04
Bagging	0.984	1.581	1.190	3.392	4.29	0.856	5.654	4.113	12.060	15.35
HistGBR	0.995	0.801	0.484	1.346	2.17	0.928	3.758	2.684	8.461	10.20
Scaled SVR	0.986	0.098	0.098	0.303	0.27	0.952	3.428	2.041	5.464	9.30
Voting	0.998	0.603	0.456	1.295	1.64	0.949	3.941	3.006	8.796	10.69
Stacking	0.999	0.590	0.472	1.465	1.60	0.988	2.487	1.493	3.903	6.75

**Table 5:** 10-Fold cross-validation performance of ML models in predicting Mu.

Model	10-Fold Cross Validation				
	R^2^	RMSE	MAE	MAPE	NRMSE
RandomForest	0.836	4.807	3.599	10.110	13.04
ExtraTrees	0.927	3.198	2.385	6.867	8.68
GradientBoosting	0.931	3.110	2.253	7.010	8.44
AdaBoost	0.837	4.790	3.698	11.016	13.00
Bagging	0.834	4.840	3.611	10.195	13.13
HistGBR	0.901	3.738	2.816	8.180	10.14
Scaled SVR	0.971	2.022	1.400	4.332	5.49
Voting	0.914	3.478	2.635	7.449	9.44
Stacking	0.977	1.784	1.288	3.865	4.84

**Table 6:** Variability of ML model performance across 10 × 10 repeated cross-validation folds for prediction of Mu.

Model	R^2^ (Mean ± SD)	RMSE (Mean ± SD)	MAE (Mean ± SD)
RF	0.864 ± 0.057	4.115 ± 0.763	3.277 ± 0.582
ET	0.923 ± 0.035	3.077 ± 0.585	2.440 ± 0.478
GB	0.949 ± 0.027	2.439 ± 0.499	1.910 ± 0.391
AB	0.846 ± 0.071	4.346 ± 0.758	3.574 ± 0.603
BG	0.875 ± 0.052	3.941 ± 0.736	3.130 ± 0.551
HGB	0.932 ± 0.036	2.872 ± 0.639	2.212 ± 0.435
SVR	0.954 ± 0.023	2.403 ± 0.688	1.727 ± 0.384
VR	0.936 ± 0.030	2.797 ± 0.539	2.240 ± 0.423
SR	0.971 ± 0.017	1.877 ± 0.489	1.400 ± 0.295

**Table 7:** Validation of model robustness via target permutation analysis.

Model	Randomized R^2^	Interpretation
SR (Stacking)	−0.71 ± 0.157	Strongest degradation → most robust
SVR	−0.670 ± 0.155	Very robust
GB	−0.622 ± 0.157	Robust
HGB	−0.583 ± 0.110	Robust
AB	−0.321 ± 0.089	Moderate
ET	−0.292 ± 0.077	Moderate
VR	−0.290 ± 0.076	Moderate
BG	−0.259 ± 0.065	Moderate
RF	−0.231 ± 0.056	Least degradation

**Table 8:** Comparison of model performance with prior studies.

Reference	Dataset Size	Train/Test Split	Model	Performance Metrics
Current study	220	80:20	SR	Training R^2^ = 0.999, RMSE = 0.590, MAE = 0.472, MAPE = 1.465, Testing R^2^ = 0.988, RMSE = 2.487, MAE = 1.493%, MAPE = 3.903%,
Ahmadi [84,85]	272	80:20	ANN	R^2^ = 0.97, MAPE = 5.8%
			Regression	R^2^ = 0.926, MAPE = 13.2%
Tran [86]	258	85:15	Regression	R^2^ = 0.95, MAE = 323.52, RMSE = 565
Guneyisi [87]	314	75:25	GEP	MAPE = 7.49%, RMSE = 228
Ipek [88]	103	75:25	GEP	R^2^ = 0.987, MAPE = 6.43%, RMSE = 85.7
Javed [89]	227	78:22	GEP	R^2^ = 0.98, MAE = 138.7, RMSE = 258
Naser [90]	1245/979	70:30	GA+	MAE = 202, RMSE = 295
			GEP	MAE = 238, RMSE = 340
Ren [91]	180	85:15	SVM	Train: R^2^ = 0.932, MAPE = 14.3%, MAE = 239, RMSE = 314 Test: R^2^ = 0.914, MAPE = 14.5%, MAE = 227, RMSE = 304
Memarzadeh [92]	646/347	85:15	GEP	R^2^ = 0.98, MAE = 242, RMSE = 384
			GEP	R^2^ = 0.98, MAE = 324, RMSE = 464
			ANN	R^2^ = 0.99, MAE = 134, RMSE = 205
			ANN	R^2^ = 0.99, MAE = 163, RMSE = 254
Megahed et al. [93]	674/396/246	80:20	SR	MAPE = 5.856%, RMSE = 552 MAPE = 5.856%, RMSE = 368.2 MAPE = 5.756%, RMSE = 194.9

## Data Availability

The data supporting the findings of this study are available from the corresponding author upon reasonable request.
